# Distinctive alterations in the mesocorticolimbic circuits in various psychiatric disorders

**DOI:** 10.1111/pcn.13542

**Published:** 2023-03-30

**Authors:** Yuko Nakamura, Takuya Ishida, Saori C. Tanaka, Yuki Mitsuyama, Satoshi Yokoyama, Hotaka Shinzato, Eri Itai, Go Okada, Yuko Kobayashi, Takahiko Kawashima, Jun Miyata, Yujiro Yoshihara, Hidehiko Takahashi, Ryuta Aoki, Motoaki Nakamura, Haruhisa Ota, Takashi Itahashi, Susumu Morita, Shintaro Kawakami, Osamu Abe, Naohiro Okada, Akira Kunimatsu, Ayumu Yamashita, Okito Yamashita, Hiroshi Imamizu, Jun Morimoto, Yasumasa Okamoto, Toshiya Murai, Ryu‐Ichiro Hashimoto, Kiyoto Kasai, Mitsuo Kawato, Shinsuke Koike

**Affiliations:** ^1^ Center for Evolutionary Cognitive Sciences, Graduate School of Art and Sciences University of Tokyo Tokyo Japan; ^2^ University of Tokyo Institute for Diversity & Adaptation of Human Mind (UTIDAHM) Tokyo Japan; ^3^ Department of Neuropsychiatry Graduate School of Wakayama Medical University Wakayama Japan; ^4^ Brain Information Communication Research Laboratory Group Advanced Telecommunications Research Institutes International (ATR) Kyoto Japan; ^5^ Information Science, Graduate School of Science and Technology Nara Institute of Science and Technology Nara Japan; ^6^ Department of Psychiatry and Neurosciences Hiroshima University Hiroshima Japan; ^7^ Department of Psychiatry, Graduate School of Medicine Kyoto University Kyoto Japan; ^8^ Department of Psychiatry and Behavioral Sciences Tokyo Medical and Dental University Tokyo Japan; ^9^ Medical Institute of Developmental Disabilities Research Showa University Tokyo Japan; ^10^ Department of Neuropsychiatry, Graduate School of Medicine University of Tokyo Tokyo Japan; ^11^ Department of Radiology, Graduate School of Medicine the University of Tokyo Tokyo Japan; ^12^ The International Research Center for Neurointelligence (WPI‐IRCN), Institutes for Advanced Study (UTIAS) University of Tokyo Tokyo Japan; ^13^ Department of Radiology International University of Health and Welfare Mita Hospital Tokyo Japan; ^14^ Department of Psychiatry Boston University School of Medicine Boston Massachusetts USA; ^15^ Center for Advanced Intelligence Project RIKEN Tokyo Japan; ^16^ Department of Psychology, Graduate School of Humanities and Sociology the University of Tokyo Tokyo Japan; ^17^ Department of Systems Science, Graduate School of Informatics Kyoto University Kyoto Japan; ^18^ Department of Language Sciences Tokyo Metropolitan University Tokyo Japan

**Keywords:** autism spectrum disorder, dynamic causal modeling, major depressive disorder, mesocorticolimbic circuits, schizophrenia

## Abstract

**Aim:**

Increasing evidence suggests that psychiatric disorders are linked to alterations in the mesocorticolimbic dopamine‐related circuits. However, the common and disease‐specific alterations remain to be examined in schizophrenia (SCZ), major depressive disorder (MDD), and autism spectrum disorder (ASD). Thus, this study aimed to examine common and disease‐specific features related to mesocorticolimbic circuits.

**Methods:**

This study included 555 participants from four institutes with five scanners: 140 individuals with SCZ (45.0% female), 127 individuals with MDD (44.9%), 119 individuals with ASD (15.1%), and 169 healthy controls (HC) (34.9%). All participants underwent resting‐state functional magnetic resonance imaging. A parametric empirical Bayes approach was adopted to compare estimated effective connectivity among groups. Intrinsic effective connectivity focusing on the mesocorticolimbic dopamine‐related circuits including the ventral tegmental area (VTA), shell and core parts of the nucleus accumbens (NAc), and medial prefrontal cortex (mPFC) were examined using a dynamic causal modeling analysis across these psychiatric disorders.

**Results:**

The excitatory shell‐to‐core connectivity was greater in all patients than in the HC group. The inhibitory shell‐to‐VTA and shell‐to‐mPFC connectivities were greater in the ASD group than in the HC, MDD, and SCZ groups. Furthermore, the VTA‐to‐core and VTA‐to‐shell connectivities were excitatory in the ASD group, while those connections were inhibitory in the HC, MDD, and SCZ groups.

**Conclusion:**

Impaired signaling in the mesocorticolimbic dopamine‐related circuits could be an underlying neuropathogenesis of various psychiatric disorders. These findings will improve the understanding of unique neural alternations of each disorder and will facilitate identification of effective therapeutic targets.

## Introduction

The mesocorticolimbic dopamine‐related circuit originates from the ventral tegmental area (VTA) and connects with various brain regions, such as the nucleus accumbens (NAc) and prefrontal cortex (PFC).^1^, ^2^, ^3^, ^4^, ^5^, ^6^, ^7^, ^8^, ^9^ From the VTA, neural projections are bidirectionally connected to the NAc.^1^, ^5^ The NAc is anatomically and functionally divided into the two subregions, shell and core.^10^ The subregions of NAc project to the PFC *via* distinctive pathways,^1^, ^3^, ^4^, ^5^, ^10^ whereas the core and shell have significant interactions within the NAc.^1^, ^11^

The mesocorticolimbic dopamine‐related circuit is involved in various functions, including reward, aversion, pain, motivated behaviors, social motivation, reinforcement learning, working memory, attention, planning, decision making, and motor control.^2^, ^3^, ^4^, ^5^, ^6^, ^7^, ^8^, ^10^, ^12^, ^13^, ^14^, ^15^ The deficits of these functions are often reported in various psychiatric disorders,^16^, ^17^, ^18^ and those dysfunctions could be linked to alterations in the mesocorticolimbic circuits.^9^, ^17^, ^18^, ^19^, ^20^, ^21^, ^22^, ^23^, ^24^, ^25^, ^26^, ^27^, ^28^, ^29^, ^30^, ^31^ For example, increased activity in the mesolimbic pathway underlies psychotic symptoms of schizophrenia (SCZ) (e.g., delusions and hallucinations) and is thus a target of pharmacological treatments.^32^ In contrast, hypodopaminergia or striatal dysfunction contribute to negative symptoms, such as flattened affect and lack of motivation.^22^, ^33^, ^34^ Blunted activity in mesocorticolimbic pathways is also related to major depressive disorder (MDD), which is characterized by anhedonia, depressed mood, and loss of interest or pleasure.^16^, ^18^, ^28^, ^29^, ^30^, ^31^, ^35^, ^36^ Deep brain stimulation, a neurosurgical procedure that uses implanted electrodes to stimulate target regions electrically, has been adapted to stimulate the NAc for patients with MDD.^37^ Further, emerging evidence shows that autism spectrum disorder (ASD) is linked to deficits in mesocorticolimbic pathways,^17^, ^24^, ^25^, ^26^, ^27^ and decreased activity in the mesocorticolimbic pathways is reported to be linked to deficits in social interactions^25^ and reward processing.^27^ Collectively, these results support that a dysregulated mesocorticolimbic circuit could underlie various psychiatric disorders, and the symptoms would have common and disease‐specific characteristics in the circuit.

Neuroimaging studies have revealed that disruptions in the regions focused on mesocorticolimbic circuit are linked to various psychiatric disorders.^36^, ^37^, ^38^, ^39^, ^40^ SCZ is associated with a reduced structural connection between NAc and PFC^41^ and to increased structural connection between VTA‐PFC and NAc‐PFC. In addition, such increased structural NAc‐PFC (medial orbitofrontal cortex) connection was negatively correlated with negative symptoms of SCZ.^39^ Moreover, increased NAc‐VTA functional connectivity on resting‐state functional magnetic resonance imaging (rs‐fMRI) is associated with auditory or visual hallucinations in participants with SCZ.^42^ Compared to healthy controls (HCs), participants with SCZ showed higher ventral striatum response to reward stimuli and more impaired functional coupling of the ventral striatum with the PFC.^43^ For MDD, dysregulated VTA, NAc, and PFC activities during reward tasks are related with anhedonia.^36^ Patients with MDD show reduced mean fractional anisotropy in the medial forebrain bundle, which connects the VTA with the NAc; medial and lateral orbitofrontal cortex; and the dorsolateral prefrontal cortex.^44^ Decreased functional connectivity of VTA with PFC at rest is reported in MDD patients.^40^ In addition, VTA‐PFC functional connectivity during music listening is negatively associated with anhedonia in these patients.^45^ Furthermore, children with ASD show lower NAc‐VTA structural connections, which are related to severe social interaction impairments.^38^ Additionally, children with ASD show decreased functional connectivity between the NAc and the VTA during face‐related stimuli processing and social stimuli processing, which is associated with more severe social interaction impairments.^38^

The shell and core NAc have distinct functions. The shell is linked to mediate the reinforcing properties of novelty, feeding behavior, unconditioned reward‐seeking behaviors, rewarding substances, and drug relapse, whereas the core is suspected to play a crucial role in spatial learning, conditioned responses, responses to motivational stimuli, guiding behavior toward a specific goal, and impulsive choices, likely operating in tandem with the anterior cingulate *via* a corticostriatal circuit.^46^, ^47^ In addition, distinct connections with each accumbens subregion provide different functions.^48^ For instance, the prefrontal cortex‐accumbens shell, but not the accumbens core, connection is related to response to novelty,^49^, ^50^ whereas the prefrontal cortex‐accumbens core, but not the accumbens shell, is related to general reward.^50^ Furthermore, much of the afferent innervation of the shell derives from cortical and subcortical structures relatively segregated from those projecting strongly to the core, whereas the outputs from the shell and core terminate in distinct structures and subregions.^48^ Thus, distinct connections with each accumbens subregion could be involved in different functions. Therefore, each subregion would be uniquely associated with psychiatric disorders. According to an rs‐fMRI study, the functional connectivity map of the core of NAc displayed different associations with the severity of anhedonia in patients with MDD than did that of the shell.^51^ However, the roles of each subregion in various psychiatric disorders are still unclear. Our previous rs‐fMRI study examined the differences and similarities in VTA‐related resting‐state functional connectivity, which are supposedly associated with the mesocorticolimbic circuit, across various psychiatric disorders, including SCZ, MDD, and bipolar disorder.^40^ The VTA‐PFC (the medial superior frontal cortex) connectivity was lower in patients with MDD and bipolar disorder than that in HCs and those with SCZ; nonetheless, there was no altered VTA‐NAc connectivity.^40^ Therefore, we conducted an rs‐fMRI study to examine the detailed differences or similarities in the mesocorticolimbic circuits across various psychiatric disorders (MDD, ASD, and SCZ) and HCs using a dynamic causal modeling (DCM) analysis, including the VTA, the core and shell of NAc, and medial PFC (mPFC) as regions of interests (ROIs). Anatomically, the mPFC bidirectionally projects to the VTA and is connected to the core and shell of the NAc.^1^, ^4^ The dopamine neurons in the VTA and their targets in the NAc and mPFC are often considered the mesocorticolimbic “reward circuit,” which responds to rewards and aversive stimuli.^2^ In the mesocorticolimbic circuit, the mPFC is implicated in a vast array of processes, including decision‐making, working memory, stimulus discrimination, stress responses, and emotional and behavioral control, and possibly associated with pathologies of various neuropsychiatric disorders.^3^, ^7^ Therefore, we selected mPFC as the ROI among the many PFC regions.

A DCM analysis refers to a method for investigating the causal inter‐regional interactions among the ROIs.^52^ A DCM analysis was originally developed for task‐based fMRI studies; however, the same state‐space modeling can be used to explain the complex cross spectra obtained from resting‐state fMRI studies. This allows functional connectivity to be explained in terms of directed effective connectivity.^53^, ^54^ Crucially, this includes the recurrent or self‐connectivity of individual regions or nodes that reflect their excitability or sensitivity to its inputs.^55^ The importance of directed connectivity lies in being able to quantify functional asymmetries in strength and parity (i.e., inhibitory *versus* excitatory) in reciprocal connections. Despite the failure of rs‐fMRI to directly measure neurotransmitter changes, resting‐state functional connectivity approximately reflects the neurotransmitter dynamics.^56^ Thus, we can assume that the hemodynamic responses reflect overall synaptic activity under the model of neuronal dynamics afforded by a DCM analysis. We hypothesized that, in the brain regions related to the mesocorticolimbic circuits, SCZ would be associated with altered effective connectivity,^39^, ^41^, ^42^, ^43^ and MDD and ASD would be associated with decreased or inhibitory connectivity.^25^, ^27^, ^36^, ^37^, ^38^, ^40^, ^45^

## Methods

### Study design and participants

Total 555 rs‐fMR images were analyzed from the database of the Japanese Strategic Research Program for the Promotion of Brain Science (SRPBS) Decoded neurofeedback (DecNef) Consortium (https://bicr.atr.jp/decnefpro/),^57^, ^58^ and additional brain images scanned in the Department of Psychiatry, The University of Tokyo (Table 1, Supplemental Methods and Materials in Appendix S1). The detailed inclusion and exclusion criteria have been previously described.^57^ This study was approved by the appropriate institutional review boards.^57^ All participants provided written informed consent.

**Table 1:** Participant characteristics by group

	HC (n = 169)	SCZ (n = 140)	MDD (n = 127)	ASD (n = 119)	*p*‐value^†^
Scanner					
COI	40	0	49	0	
KTT	30	37	0	0	
KUT	30	40	11	0	
SWA	33	18	0	114	
UTO	36	45	67	5	
Sex (male/female)	110/59	77/63	70/57	101/18	<0.001
Age (years)	35.4 ± 10.1 (19.0–58.0)	36.2 ± 10.5 (16.0–56.0)	38.8 ± 9.7 (18.0–59.0)	31.9 ± 8.0 (20.0–54.0)	<0.001
Handedness (right/left)	158/11	126/13^‡^	120/7	107/12	0.45
Medications					
Antipsychotics (mg/day or n)	N.A.	574.32 ± 459.31^§^	60.87 ± 114.33^d^	14 (11.8%)	
Antidepressants (mg/day or n)	N.A.	N.A.	135.07 ± 132.16^¶^	21 (17.6%)	
Anti‐anxiety and sleep‐inducing drug (n)	N.A.	N.A.	N.A.	30 (25.2%)	

Abbreviations: ASD, Autism spectrum disorder; COI, Siemens Verio scanner at the Center of Innovation in Hiroshima University; HC, healthy controls; KTT, Siemens Trio scanner at Kyoto University; KUT, Siemens TimTrio scanner at Kyoto University; MDD, Major depressive disorder; SCZ, schizophrenia; SWA, Siemens Verio scanner at Showa University; UTO, GE MR750W scanner at The University of Tokyo Hospital.

^†^ Differences in sex and handedness are tested using a chi‐squared test, and differences in age are tested using a Kruskal–Wallis rank sum test.

^‡^ Data missing for one participant.

^§^ Chlorpromazine equivalent dose for participants scanned with KTT, KUT, and UTO, but not SWA. Data missing for two participants.

^¶^ Chlorpromazine and imipramine equivalent dose for participants scanned with KUT and UTO, but not COI.

### Clinical assessment

The severity of psychiatric symptoms was assessed using the Japanese version of the Beck Depression Inventory‐II^59^, ^60^ for MDD, the Positive and Negative Syndrome Scale^61^ for SCZ, and the Autism‐Spectrum Quotient Test (AQ)^62^, ^63^, ^64^ for ASD, which were available from all institutes in the database (Table S1, Supplemental Methods and Materials in Appendix S1).

### rs‐fMRI data acquisition

rs‐fMRI data were acquired using five different scanners. (Table 2, Table S2, Supplemental Methods and Materials in Appendix S1). We instructed the participants to relax but not to sleep during scanning, and to focus on the central crosshair mark.

**Table 2:** Clinical assessment settings and image acquisition parameters at each scanner

	COI	KTT	KUT	SWA	UTO
Clinical assessment					
Diagnosis	DSM‐IV‐TR or DSM‐5	DSM‐IV‐TR (using SCID)	DSM‐IV‐TR (using SCID)	DSM‐IV‐TR (using SCID)	DSM‐IV‐TR
Examiner	Experienced psychiatrists	Experienced psychiatrists	Experienced psychiatrists	Experienced psychiatrists	Experienced psychiatrists
Exclusion criteria					
History of neurological disorders	Excluded	Excluded	Excluded	Excluded	Excluded
History of head trauma with accompanying loss of consciousness >5 min		Excluded	Excluded	Excluded	Excluded
Alcohol or illegal drug abuse	Excluded	Excluded	Excluded	Excluded	Excluded
Image acquisition parameters					
MRI scanner	Siemens verio	Siemens Trio	Siemens TimTrio	Siemens verio	GE MR750w
Magnetic field strength	3.0 T	3.0 T	3.0 T	3.0 T	3.0 T
Head coil channels	12	8	32	12	24
TR (ms)	2500	2.000	2500	2.500	2500
TE (ms)	30	30	30	30	30
Total scan time (min:s)	10:00	6:00	10:00	10:00	10:00
Phase encoding direction	AP	AP	PA	PA	PA

Abbreviations: AP, anterior–posterior; COI, Siemens Verio scanner at the Center of Innovation in Hiroshima University; DSM‐5, DSM Fifth Edition; DSM‐IV‐TR, the Diagnostic and Statistical Manual, Fourth Edition (text revision); KTT, a Siemens Trio scanner at Kyoto University; KUT, a Siemens TimTrio scanner at Kyoto University; PA, posterior–anterior; SCID, the Structured Clinical Interview for DSM‐IV Axis I Disorders; SWA, a Siemens Verio scanner at Showa University; TE, echo time; TR, repetition time; UTO, GE MR750W scanner at The University of Tokyo Hospital.

### Dynamic causal model for resting state fMRI time series

#### Image preprocessing

Image preprocessing was performed using Statistical Parametric Mapping (SPM12, v7771; Wellcome Department of Cognitive Neurology, London, UK) in Matlab R2019b (Mathworks, Natick, MA, USA). Conventional preprocessing was performed (detailed procedures were described in the Supplemental Methods and Materials in Appendix S1).

#### Regions of interest

**Fig. 1:**
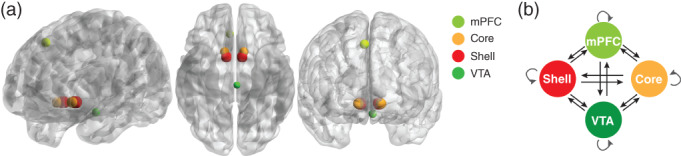
Regions of interest and the DCM model. (a) Regions of interest (ROIs) rendered on the sagittal, axial, and coronal slice of the brain. Spheres (3 mm) are created for core, shell, and VTA ROIs. A 5‐mm sphere is created for the mPFC ROI. The center of each ROI is as follows: the right core = ([*x*, *y*, *z*] = [12, 12, −12]), left core = ([*x*, *y*, *z*] = [−12, 12, −12]), right shell = ([*x*, *y*, *z*] = [12, 8, −12]), left shell = ([*x*, *y*, *z*] = [−12, 8, −12]), medial prefrontal cortex (mPFC) = ([*x*, *y*, *z*] = [6, 34, 50]), and ventral tegmental area (VTA) = ([*x*, *y*, *z*] = [−2, −20, −18]). (b) DCM models. A fully connected model containing the following 16 connectivity parameters is constructed: 12 connections between regions (black) and four recurrent self‐connections (gray). DCM, dynamic causal model; mPFC, medial prefrontal cortex; VTA, ventral tegmental area.

The VTA, core of the NAc, shell of the NAc, and mPFC masks were created as the ROIs (Fig. 1a, Supplemental Methods and Materials in Appendix S1). Subsequently, the averaged first principal component of the time‐series from all voxels included in the ROIs was extracted for the DCM analysis. To avoid time‐series extraction from the non‐brain region, each ROI mask was multiplied with the binarized whole brain mask thresholded at 0.5 of the intensity.

#### Spectral DCM analysis

To estimate connectivity among the four brain regions, spectral DCM (spDCM)^53^ was adapted for rs‐fMRI data using DCM12.5 as implemented in SPM12 v7771. spDCM analysis involves a specification of a plausible network model, which enables the estimation of the model parameters that quantify effective connectivity and regionally specific hemodynamic variables.^53^, ^54^ In DCM, self‐connections only exert an inhibitory influence on each region included in the model.^55^ Therefore, such inhibitory self‐connections reflect the rate of decay of neuronal activity in each region, where a greater self‐inhibition indicates that a region is less sensitive to its inputs.^52^ Model specification comprised the selection of the ROIs and definition of the model space with respect to connectivity between regions. A fully connected model was constructed for each participant (Fig. 1b). Subsequently, DCMs were estimated using spectral DCM, which fits the complex cross‐spectral density using a parameterized power‐law model of endogenous neural fluctuations.^54^ Next, model inversion was conducted based on standard variational Laplace procedures.^65^ This Bayesian inference method uses Free Energy as a proxy for (log) model evidence, while optimizing the posterior density under Laplace approximation of model parameters.

#### Parametric empirical bayes estimation

We conducted group‐level inference for spDCM using an empirical Bayesian approach with SPM12.^66^, ^67^ Parametric empirical Bayes (PEB) consists of Bayesian model reduction, searching over nested models, and comparison of effective connectivity parameters^68^ (Supplemental Methods and Materials in Appendix S1). Then, we performed a general linear model (GLM) analysis to determine the difference or commonality in estimated connection strengths between groups. We set up three GLMs. The first GLM had the following contrasts: mean of all participants, HC *vs*. all patient groups (MDD, SCZ, and ASD), MDD *vs*. SCZ, and SCZ *vs*. ASD. The second GLM had the following contrasts: mean of all participants, HC *vs*. all patient groups, MDD *vs*. SCZ, and MDD *vs*. ASD. The third GLM had the following contrasts: HC *vs*. MDD, HC *vs*. SCZ, and HC *vs*. ASD.

Significant group differences in age (Kruskal–Wallis *χ* ^2^ = 32.7, *p* < 0.001, *df* = 3) (Table 1, Fig. S1a in Appendix S1) and sex (*χ* ^2^ = 32.0, *p* < 0.001, *df* = 3) were observed (Table 1). Age, sex, and scanner were included in the GLM as covariates‐of‐no‐interest to regress out the effect of these variables. Considering the strong male bias in ASD prevalence,^69^ we performed the same group comparisons in male participants (Tables S4 and S5, Fig. S1b in Appendix S1) to determine if the results from all participants were replicated in men. For this analysis, age and scanner were included into the GLMs as covariates‐of‐no‐interest.

#### Associations between estimated effective connectivity and clinical variables

To examine the potential effect of medication on connectivity that showed differences between groups, a multiple linear regression analysis was performed that included estimated connectivity parameters extracted from individual fully connected models as a dependent variable and medications as predictor variables. The significant threshold was set at uncorrected *p* < 0.05. The relationship between symptom severity and estimated effective connectivity that showed significant differences in connection between the groups was analyzed using Pearson's correlation analysis. The significant threshold was set at *p*_Bonferroni corrected_ <0.05.

## Results

### Commonality of effective connectivity across all participants

**Fig. 2:**
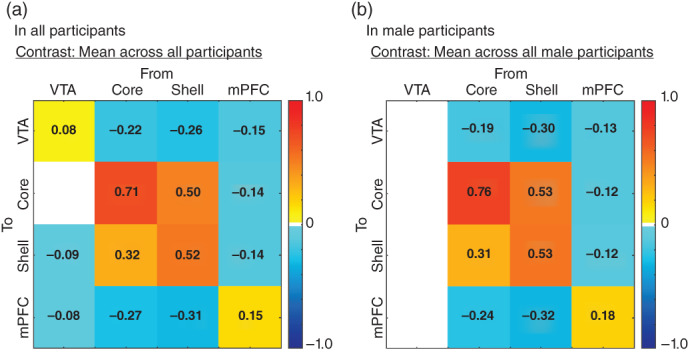
Intrinsic connectivity matrix reflecting effective connectivity across ROIs in all participants (a) and in male participants (b). Only connections with a posterior probability greater than 95% are displayed. Color gradient in matrixes indicates the intrinsic connectivity strengths. Color bars reflect positive or negative values for the intrinsic connections. HC, healthy controls; SCZ, schizophrenia; MDD, major depressive disorder; ASD, autism spectrum disorders; VTA, ventral tegmental area; mPFC, medial prefrontal cortex.

In all participants, excitatory connectivity was found in the shell‐to‐core and core‐to‐shell connectivities. Whereas, inhibitory connectivity was found in the VTA‐to‐VTA, core‐to‐core, shell‐to‐shell, mPFC‐to‐mPFC, core‐to‐VTA, shell‐to‐VTA, mPFC‐to‐VTA, mPFC‐to‐core, VTA‐to‐shell, mPFC‐to‐shell, VTA‐to‐mPFC, core‐to‐mPFC, and shell‐to‐mPFC connectivities (Fig. 2a, Fig. S2a in Appendix S1).

In male participants, excitatory connectivity was found in the shell‐to‐core and core‐to‐shell connectivities. Whereas, inhibitory connectivity was found in the following connectivity: core‐to‐core, shell‐to‐shell, mPFC‐to‐mPFC, core‐to‐VTA, shell‐to‐VTA, mPFC‐to‐VTA, mPFC‐to‐core, mPFC‐to‐shell, core‐to‐mPFC, and shell‐to‐mPFC (Fig. 2b, Fig. S2b in Appendix S1). Inhibitory connectivity in the VTA‐to‐VTA, VTA‐to‐shell, and VTA‐to‐mPFC in all participants was not replicated in male participants.

### Group differences in effective connectivity across groups

**Fig. 3:**
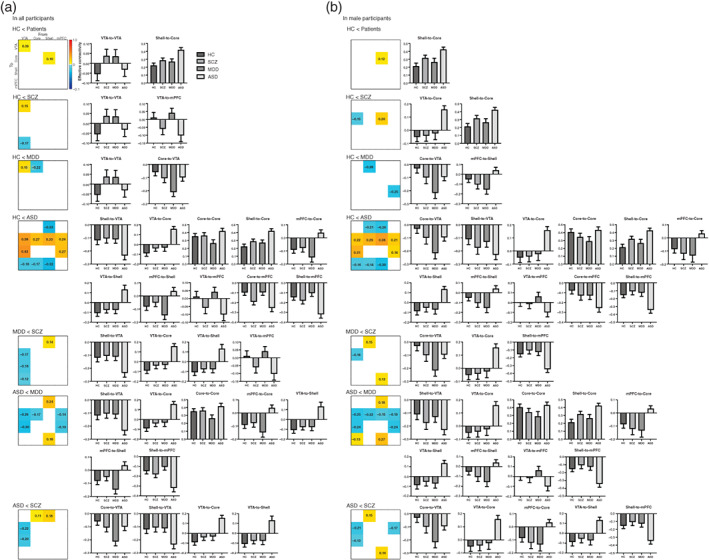
Intrinsic connectivity matrix reflecting group differences in effective connectivity across ROIs in all participants (a) and in male participants (b). Only connections with a posterior probability greater than 95% are displayed. Color gradient in matrixes indicates connection strengths as a difference between two groups. Color bars reflect positive or negative values for differences in those connections. Bar graphs indicate estimated effective connectivity that show significant differences between groups for all and male participants. ASD, autism spectrum disorders; HC, healthy controls; MDD, major depressive disorder; mPFC, medial prefrontal cortex; SCZ, schizophrenia; VTA, ventral tegmental area.

From the HC *vs*. all patients contrast, intergroup differences were observed in the VTA‐to‐VTA and shell‐to‐core contrasts (strong evidence posterior probability [Pp] >0.95) (Fig. 3a, Fig. S2a in Appendix S1). The VTA‐to‐VTA connectivity was less negative in the HC and ASD groups, but more negative in the SCZ and MDD groups. The excitatory shell‐to‐core connectivity was greater in all patient groups than in the HC group.

The inhibitory VTA‐to‐VTA connectivity was greater in the SCZ group than in the HC group. The VTA‐to‐mPFC connectivity was inhibitory in the SCZ group, but excitatory in the HC. The inhibitory VTA‐to‐VTA and core‐to‐VTA connectivities were greater in the MDD group than in the HC group. The inhibitory shell‐to‐VTA, core‐to‐core, core‐to‐mPFC, and shell‐to‐mPFC connectivities were greater in the ASD group than in the HC group. The excitatory shell‐to‐core connectivity was also greater in the ASD group than in the HC group. The VTA‐to‐core, mPFC‐to‐core, VTA‐to‐shell, and mPFC‐to‐shell connectivities were excitatory in the ASD group, but inhibitory in the HC group. Conversely, the VTA‐to‐mPFC connectivity was inhibitory in the ASD group, but excitatory in the HC group (Fig. 3a, Fig. S2a in Appendix S1).

Within disease contrasts, the inhibitory shell‐to‐VTA connectivity was lesser, but the inhibitory VTA‐to‐core and VTA‐to‐shell connectivities were greater, in the SCZ group than in the MDD group. The VTA‐to‐mPFC connectivity was inhibitory in the SCZ group, but excitatory in the MDD group. The inhibitory shell‐to‐VTA, core‐to‐core, and shell‐to‐mPFC connectivities were greater in the ASD group than in the MDD group. The VTA‐to‐core, mPFC‐to‐core, VTA‐to‐shell, and mPFC‐to‐shell connectivities were excitatory in the ASD group, but inhibitory in the MDD group. The inhibitory core‐to‐VTA and shell‐to‐VTA connectivities were lesser in the SCZ group than in the ASD group. The VTA‐to‐core and VTA‐to‐shell connectivities were inhibitory in the SCZ group, but excitatory in the ASD group (Fig. 3a, Fig. S2a in Appendix S1).

Among the male participants, an intergroup difference was observed in the shell‐to‐core contrasts from the HC *vs*. all patients contrast. Unlike in the HC group, all patient groups showed a greater excitatory shell‐to‐core connectivity (Fig. 3b, Fig. S2b in Appendix S1).

The VTA‐to‐core connectivity was more inhibitory in the SCZ group as compared to in the HC group. The excitatory shell‐to‐core connectivity was greater in the SCZ group than in the HC group. The inhibitory core‐to‐VTA and mPFC‐to‐VTA connectivities were greater in the MDD group than in the HC group. The inhibitory core‐to‐VTA, shell‐to‐VTA, core‐to‐core, core‐to‐mPFC, and shell‐to‐mPFC connectivities were greater in the ASD group than in the HC group. The excitatory shell‐to‐core connectivity was greater in the ASD group than in the HC group. The VTA‐to‐core, mPFC‐to‐core, VTA‐to‐shell, and mPFC‐to‐shell connectivities were excitatory in the ASD group, but inhibitory in the HC group. Conversely, the VTA‐to‐mPFC connectivity was inhibitory in the ASD group, but excitatory in the HC group (Fig. 3b, Fig. S2b in Appendix S1).

Within the disease contrasts, the inhibitory core‐to‐VTA and shell‐to‐mPFC connectivities were lesser in the SCZ group than in the MDD group; the inhibitory VTA‐to‐core connectivity was greater in the SCZ group than in the MDD group. The inhibitory shell‐to‐VTA, core‐to‐core, and shell‐to‐mPFC connectivities were greater in the ASD group than in the MDD group. The excitatory shell‐to‐core connectivity was greater in the ASD group than in the MDD group. The VTA‐to‐core, mPFC‐to‐core, VTA‐to‐shell, and mPFC‐to‐shell connectivities were excitatory in the ASD group, but inhibitory in the MDD group. Conversely, the VTA‐to‐mPFC connectivity was inhibitory in the ASD group, but excitatory in the MDD group. The core‐to‐VTA and shell‐to‐mPFC connectivities were less inhibitory in the SCZ group than in the ASD group. The VTA‐to‐core, mPFC‐to‐core, and VTA‐to‐shell connectives were inhibitory in the SCZ group, but excitatory in the ASD group (Fig. 3b, Fig. S2b in Appendix S1).

For all participants and all male participants, there were several similar differences in the effective shell‐to‐core connectivity in the HC *vs*. all patients contrast; the effective core‐to‐VTA connectivity in the MDD *vs*. HC contrast; the effective shell‐to‐VTA, VTA‐to‐core, core‐to‐core, shell‐to‐core, mPFC‐to‐core, VTA‐to‐shell, mPFC‐to‐shell, VTA‐to‐mPFC, core‐to‐mPFC, and shell‐to‐mPFC connectivities in the ASD *vs*. HC contrast; the effective VTA‐to‐core connectivity in the MDD *vs*. SCZ contrast; the effective shell‐to‐VTA, VTA‐to‐core, core‐to‐core, mPFC‐to‐core, VTA‐to‐shell, mPFC‐to‐shell, and shell‐to‐mPFC connectivities in the ASD *vs*. MDD contrast; and the effective core‐to‐VTA, VTA‐to‐core, and VTA‐to‐shell connectivities in the ASD *vs*. SCZ contrast (Fig. 3).

### Associations between estimated effective connectivity and clinical variables

**Fig. 4:**
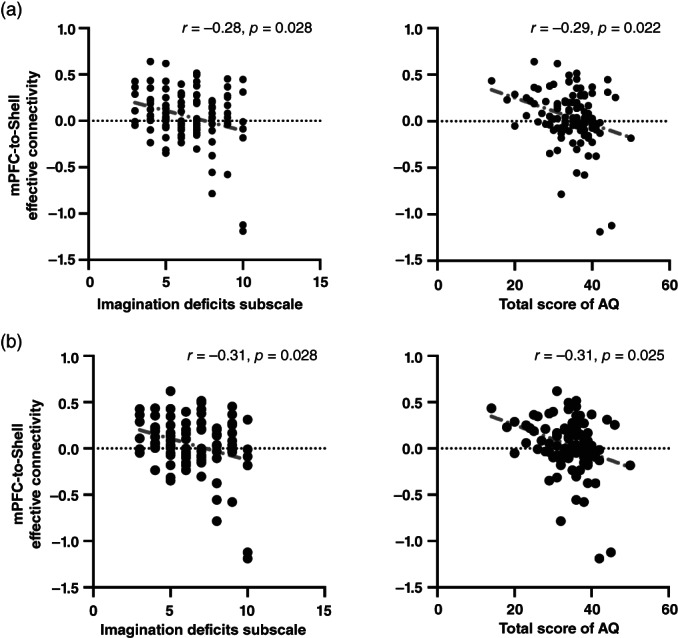
Associations between effective connectivity of the PFC‐to‐shell and clinical assessments in the ASD group (a) and in the male ASD patients (b). AQ, the Autism‐spectrum Quotient test; ASD, autism spectrum disorders; mPFC, medial prefrontal cortex.

No connectivity showed significant associations with any medication equivalent doses (SCZ: *ps* >0.06, MDD: *ps* >0.07) or any medication usage (ASD: *ps* >0.11), except for the shell‐to‐VTA connectivity, which showed a positive association with the antidepressant equivalent dose in the MDD group (*p*_uncorrected_ = 0.051, β = 0.286). After Bonferroni correction, this association was not statistically significant (*p*_Bonferroni‐corrected_ = 0.136). For ASD, effective connectivity of the mPFC‐to‐shell was negatively correlated with scores of the imagination deficits subscale of AQ and total AQ (total: *r* = −0.28, *p*_Bonferroni corrected_ = 0.028 and *r* = −0.29, *p*_Bonferroni corrected_ = 0.022; male: *r* = −0.31, *p*_Bonferroni corrected_ = 0.028 and *r* = −0.31, *p*_Bonferroni corrected_ = 0.025, respectively) (Fig. 4). There was no significant association between effective connectivity and clinical assessment in any other groups.

## Discussion

This study found that compared with the HC group, the SCZ, MDD, and ASD groups showed a greater excitatory shell‐to‐core connectivity. Compared with the MDD, SCZ, and HC groups, the ASD showed greater inhibitory shell‐to‐VTA and shell‐to‐mPFC connectivities. Conversely, the VTA‐to‐core and VTA‐to‐shell connectivities were excitatory in the ASD group, but inhibitory in the MDD, SCZ, and HC groups. These results were also found in male participants. Further, the mPFC‐to‐shell connectivity was negatively associated with the imagination deficit subscale of AQ and total AQ scores in ASD.

We examined the commonality of effective connectivity across all participants. In the current sample, inter NAc connectivities were excitatory. Animal studies suggested that, in the NAc, direct or indirect inter subregion connections were observed.^1^, ^11^ Some of these connections were mediated by glutamate and dopamine,^1^, ^11^ and such connections could be excitatory. Whereas core‐to‐VTA, shell‐to‐VTA, mPFC‐to‐VTA, mPFC‐to‐core, mPFC‐to‐shell, core‐to‐mPFC, core‐to‐core, shell‐to‐shell, mPFC‐to‐mPFC, and shell‐to‐mPFC connectivities were inhibitory. The mesocorticolimbic circuits including the VTA, NAc, and mPFC are composed of complex synaptic inputs from excitatory, inhibitory, and modulatory neurons.^1^, ^70^ Further, with the dopamine neurons, the GABAergic neurons and glutamatergic neurons in the VTA display diversity in the mesocorticolimbic circuits.^1^, ^2^ For example, glutamatergic inputs from the mPFC synapse onto VTA dopamine neurons that project back to the mPFC, but not dopamine neurons that project to the NAc.^2^ Because of such heterogeneity of neurons in the mesocorticolimbic circuits, the excitatory, inhibitory, or modulatory pathways remain to be clarified. The current results could be clues to address which pathways would be excitatory, inhibitory, or modulatory at rest in humans.

Compared with the HC group, other patient groups showed a greater excitatory shell‐to‐core connectivity. The core and shell of the NAc could connect with each other directly and indirectly.^1^, ^11^ It has been hypothesized that the shell and core share a feed‐forward functional connectivity; in such shell–core connections, neural information flows rather directly from the shell to the core.^48^ The shell–core neural connection is involved in various functions, such as reward, motivation, and stress processing^48^; deficits in these functions are often reported in various psychiatric disorders.^16^, ^17^, ^18^ For example, a postmortem study showed that SCZ is associated with increased excitatory input in the core, but not in the shell.^71^ Therefore, the increased excitatory shell‐to‐core connectivity would lead to an imbalanced feed‐forward neural information flow across the shell–core connection, and be associated with psychiatric symptoms.

Compared with the MDD, SCZ, and HC groups, the ASD group showed greater inhibitory shell‐to‐VTA and shell‐to‐mPFC connectivities. It was previously reported that a decreased NAc‐VTA connectivity is associated with ASD.^38^ In addition, the NAc and PFC are involved in the social brain, which is the brain structures traditionally associated with social cognitive processes, and the NAc is required for social reward or social behavior in animals.^17^, ^27^ Patients with ASD have reduced dopamine release in the prefrontal cortical area and diminished responsiveness of NAc.^25^ In sum, impaired NAc related connectivity could be responsible for aberrant social behaviors in ASD.

Conversely, we hypothesized that the ASD group would display inhibitory or lower excitatory connectivity in the mesocorticolimbic circuits; nonetheless, our findings revealed that the VTA‐to‐core and VTA‐to‐shell connectivities were excitatory in the ASD group, but inhibitory in the MDD, SCZ, and HC groups. Given that the therapeutic efficacy of dopamine receptor blockers in alleviating abnormal social behaviors in children with ASD,^7^ some features of ASD could be attributed to elevated dopamine neuron activity. Furthermore, animal studies showed that ASD‐related gene mutation could be related to less inhibition of ventral or dorsal striatum.^72^ Therefore, increased shell or core activity due to excitatory projections to NAc could be connected to ASD; nevertheless, further studies are warranted to validate this finding.

Although MDD and ASD are linked to decreased activity related to the mesocorticolimbic circuits,^25^, ^27^, ^36^, ^37^, ^38^, ^40^, ^45^ the current study found differences in impaired connectivity between them. The ASD group showed greater inhibitory shell‐to‐mPFC connectivity than the MDD group. Further, estimated parameters of mPFC‐to‐shell connectivity was associated with imagination deficits in the ASD group. Therefore, impaired NAc‐mPFC connectivity might be an underlying neuropathology of ASD. Animal studies showed that projection from the PFC to NAc was involved in reward learning and seeking^3^ and that the shell is related to novelty.^50^ Thus, mPFC‐to‐shell connection would, by extension, be linked to novelty. The finding of aberrant connectivity with the shell of the NAc speaks to deficits in responding to novelty, particularly in the ASD group, showing a correlation with deficits in imagination. This is a notable finding given the known involvement of dopamine in the encoding of uncertainty and novelty and the close relationship between novelty and surprise.^73^, ^74^, ^75^ This relationship can be seen at several levels, ranging from the deployment of saccadic eye movements to the choice of behavior.^76^ The correlation between connectivity and imagination lends a construct validity to the estimates of effective connectivity and may provide a fundamental link between novelty and imagination. In the context of planning an inference,^77^, ^78^, ^79^ imagination can be construed as imagining the consequences of action and evaluating them in terms of their epistemic or explanatory value; namely, the novelty of the outcomes. This is consistent with theories of autism that emphasize a lack of central coherence and difficulties disengaging from the sensorium (e.g., aversion to unpredictable and novel environments).^80^, ^81^, ^82^

This study showed that compared with the HC group, the MDD group showed a greater inhibitory core‐to‐VTA connectivity. However, we did not replicate our previous finding of a decreased functional VTA‐mPFC connectivity.^40^ Differences in the demographics of the participants, MRI machines, and analysis methods between our previous and current studies could explain these inconsistent findings. Furthermore, some neurological mechanisms may underlie the greater inhibitory core‐to‐VTA connectivity observed in the MDD group. A part of the core‐to‐VTA connectivity is inhibitory.^1^ An increased inhibitory core‐to‐VTA connectivity could lead to blunted activity in the mesocorticolimbic pathways, which is related to MDD.^16^, ^18^, ^28^, ^29^, ^30^, ^31^, ^35^, ^36^ Therefore, an increased effective inhibitory core‐to‐VTA connectivity could play a role in MDD.

Among all participants, the inhibitory VTA‐to‐VTA connectivity was greater in the SCZ group than in the HC group. The VTA‐to‐core connectivity was inhibitory in the SCZ group, but excitatory in the HC group. Among all male participants, the inhibitory VTA‐to‐core connectivity was lesser and the excitatory shell‐to‐core connectivity was greater in the SCZ group than in the HC group. Thus, unlike in other group comparisons (e.g., HC *vs*. all patients or ASD *vs*. MDD), any results from the SCZ *vs*. HC contrast in all participants were not replicated in all male participants. However, in both samples, SCZ was related to a blunted response to inputs in the VTA. Increased activity in the mesolimbic pathway in SCZ is a major target for pharmacological treatments,^32^ and in the current study, most patients with SCZ were on medications. Their symptoms were mostly stable during scanning, indicating that activity in the mesocorticolimbic circuits was stabilized. In contrast, antipsychotic medications offer little benefit against negative symptoms,^34^ which are associated with hypodopaminergia.^22^, ^33^, ^34^ Despite the patients with SCZ being under medication, hypodopaminergia persisted in the VTA connections and remained associated with the negative symptoms of SCZ.

This study has some limitations. First, given that most SCZ and MDD participants were medicated, we could not exclude the effect of medications on results. Second, because this study aimed to examine mesocorticolimbic circuits, we included four regions, namely, the VTA, shell of the NAc, core of the NAc, and mPFC. However, the mPFC is a relatively vast region and includes various subregions. In addition, other brain regions are involved in neural substrates of psychiatric disorders. Future studies should include other brain regions, such as the anterior cingulate cortex, which is likely to be linked to anhedonia.^16^, ^36^ Third, dopamine activity was not directly measured in the current study although resting‐state functional connectivity can approximately reflect the neurotransmitter dynamics.^56^ Therefore, in the future, PET studies or pharmacological studies should be performed to measure or manipulate dopamine activities in various psychiatric disorders. Fourth, although we controlled for the effect of differences in scanner and protocol at the group level analysis, they might still affect the results of group comparisons. In the future, data harmonization methods, such as ComBat,^83^ should be developed for DCM analysis.

In summary, the current study examined similarities or disease‐specific differences related to mesocorticolimbic circuits across patients with SCZ, MDD, and ASD. Compared with the HC group, the SCZ, MDD, and ASD groups showed a greater excitatory shell‐to‐core connectivity. This may indicate that an imbalanced feed‐forward neural information flow in the shell–core connection could be associated with psychological symptoms. The inhibitory shell‐to‐VTA and shell‐to‐mPFC connectivities were greater in the ASD group than in the MDD, SCZ, or HC groups. Conversely, the VTA‐to‐core and VTA‐to‐shell connectivities were excitatory in the ASD group, but inhibitory in the MDD, SCZ, and HC groups. Thus, disrupted NAc‐related connectivities could be an underlying pathology of ASD. In addition, the MDD and SCZ groups showed elevated inhibitory inputs in the VTA, which could be connected to hypodopaminergia in the mesocorticolimbic pathways. Collectively, each disorder is related to unique alternations in the mesocorticolimbic dopamine‐related circuits, and impaired signaling in these neural circuits could be an underlying neuropathogenesis of those psychiatric disorders. These findings provided novel insights for identifying critical targets for the effective treatment of each psychiatric disorder.

## Author contributions

YN, SK, and TI conceptualized and designed the study. SCT, YM, SY, HS, EI, GO, YK, TK, JM, YY, HT, RA, MN, HO, TI, SM, SK, OA, NO, AK, AY, OY, HI, JM, YO, TM, RIH, KK, and MK collected data. YN and TI analyzed the data. YN and SK drafted and reviewed the manuscript. All authors had final approval of the published manuscript.

## Funding information

This research was supported in part by AMED (Grant Number JP18dm0307001, JP18dm0307004, JP18dm0307008, JP19dm0207069, and JP21uk1024002), JST Moonshot R&D (JPMJMS2021), JSPS KAKENHI (JP20H05064, JP20KK0193, JP21H02851, JP21H05324, JP20H03596, JP21H05171, and JP21H05174), Takeda Science Foundation, and the Naito Foundation. This study was also supported by the International Research Center for Neurointelligence (WPI‐IRCN), the University of Tokyo.

## Disclosure statement

The authors declare no competing interests.

## Supporting information


**Appendix S1.**Supporting Information

## Data Availability

The data used in this study are available as part of the publicly available database of the SRPBS DecNef Consortium (https://bicr.atr.jp/decnefpro/). Please apply to the following URL (https://bicr.atr.jp/decnefpro/data/). Some MRI and clinical data are available after the ethical approval. Further inquiries can be directed to the corresponding author (S.K.).
