# Supplementary material for: Distinctive alterations in the mesocorticolimbic circuits in various psychiatric disorders
Source: Psychiatry Clin Neurosci. 2023 Mar 30;77(6):345–54. doi: 10.1111/pcn.13542 (PMC11488596; doi:10.1111/pcn.13542)
Supplement: Supplementary file 1 — Appendix S1.Supporting Information [file PCN-77-345-s001.docx]

**Distinctive alterations of the mesocorticolimbic circuits in various psychiatric disorders**

Yuko Nakamura, D.D.S., Ph.D.^1,2,*^; Takuya Ishida, M.D., Ph.D.^1,3^; Saori C. Tanaka, Ph.D.^4,5^; Yuki Mitsuyama^6^; Satoshi Yokoyama, Ph.D.^6^; Hotaka Shinzato, M.D., Ph.D.^6^; Eri Itai, M.D.^6^; Go Okada, M.D., Ph.D.^6^; Yuko Kobayashi, M.D.^7^; Takahiko Kawashima, M.D.^7^; Jun Miyata, M.D., Ph.D.^7^; Yujiro Yoshihara, M.D., Ph.D.^7^; Hidehiko Takahashi, M.D., Ph.D.^8^; Ryuta Aoki, Ph.D.^9^; Motoaki Nakamura, M.D., Ph.D.^9^; Haruhisa Ota, M.D., Ph.D.^9^; Takashi Itahashi, Ph.D.^9^; Susumu Morita, M.D., Ph.D.^10^; Shintaro Kawakami, M.D., Ph.D.^10^; Osamu Abe, M.D., Ph.D.^11^; Naohiro Okada, M.D., Ph.D.^12^; Akira Kunimatsu, M.D., Ph.D.^13^; Ayumu Yamashita, Ph.D.^4,14^; Okito Yamashita, Ph.D.^4,15^; Hiroshi Imamizu, Ph.D.^4,16^; Jun Morimoto, Ph.D.^4,17^; Yasumasa Okamoto, M.D., Ph.D.^6^; Toshiya Murai, M.D., Ph.D.^7^; Ryu-Ichiro Hashimoto, Ph.D.^9,18^; Kiyoto Kasai, M.D., Ph.D.^1,2,10,12^; Mitsuo Kawato, Ph.D.^4,15^; Shinsuke Koike, M.D., Ph.D.^1,2,12,*^

**Supplementary materials**

Contents

**Supplemental Methods and Materials**

**Table S1. Clinical assessment per group**

**Table S2. Image acquisition parameters per procedure**

**Table S3. Head motion in each group**

**Table S4. Characteristics of male participants**

**Table S5. Clinical assessment in male participants per group**

**Table S6. Head motion in male participants**

**Figure S1. Age of all participants (A) and male participants (B)**

**Figure S2. Effective connectivity of all participants (A) and male participants (B)**

**References**

# **Supplemental Methods and Materials**

# **Study design and participants**

# Total 555 rs-fMR images were analyzed from the database of the Japanese Strategic Research Program for the Promotion of Brain Science (SRPBS) Decoded neurofeedback (DecNef) Consortium (https://bicr.atr.jp/decnefpro/) ^1, 2^, and additional brain images scanned in the Department of Psychiatry, The University of Tokyo. The datasets were obtained from four institutes with five scanners. They included data of participants with psychiatric diseases and field map correction images for a geometric distortion correction study (140 patients with SCZ from four scanners, 127 patients with MDD from three scanners, 119 patients with ASD from two scanners, and 169 HCs matched for the age, sex, and handedness per scanner from five scanners). All participants were aged between 18 years to 60 years when they underwent brain scanning.

# **Clinical assessment**

# The severity of psychiatric symptoms was assessed using the Japanese version of the Beck Depression Inventory-II 59, 60 for MDD, the Positive and Negative Syndrome Scale 61 for SCZ, and the Autism-Spectrum Quotient Test (AQ) 62-64 for ASD, which were available from all institutes in the database. Medication use information was available for 61% of MDD participants (78/127, chlorpromazine and imipramine equivalent doses), 86% of SCZ participants (120/140, chlorpromazine dose), and all participants with ASD (antipsychotics, antidepressant, and antianxiety and sleep-inducing drug use).

# **rs-fMRI data acquisition**

# rs-fMRI data were acquired using five different scanners: a Siemens Verio scanner at the Center of Innovation in Hiroshima University (COI), a Siemens Trio scanner at Kyoto University (KTT), a Siemens TimTrio scanner at Kyoto University (KUT), a Siemens Verio scanner at Showa University (SWA), and a GE MR750W scanner at The University of Tokyo Hospital (UTO). Some of the acquisition parameters, such as 3.2 mm to 4.0 mm slice thickness and 2,000 ms to 2,500 ms TR, were determined based on the SRPBS project. We instructed the participants to relax but not to sleep during scanning, and to focus on the central crosshair mark. Each participant underwent a single rs-fMRI session for 10 min and 6 min with four scanners and KTT, respectively.

# **Dynamic causal model for resting state fMRI time series**

# *Image preprocessing*

# Image preprocessing was performed using Statistical Parametric Mapping (SPM12, v7771; Wellcome Department of Cognitive Neurology, London, UK) in Matlab R2019b (Mathworks, Natick, MA, USA). Conventional preprocessing was performed (Supplemental Methods and Materials). First, slice timing correction and geometric distortion correction^3^ were conducted for functional images. Then, the participant's high-resolution T1-weighted anatomical image was coregistered to their functional images. The coregistered anatomical image was processed using a unified segmentation procedure combining segmentation, bias correction, and spatial normalization into a standard template (Montreal Neurological Institute). The same normalization parameters were used to normalize the functional images. We excluded participants with an estimated head-motion exceeding 3 mm in any direction from the analysis. This is because 1 voxel size was approximately 3 × 3 × 3 mm^3^. Furthermore, several of our regions of interest were separated by small distances (in the order of a few millimeters). This called for a spatially precise region of interest definition that was not confounded by head movement (Table S3). Normalized functional images were smoothed in space with a 6-mm full-width at half-maximum 3D isotropic Gaussian kernel and high-pass filtered with a cut-off of 128 s (0.01 Hz) to remove low-frequency drifts. Furthermore, we calculated the derivative or root mean square variance over voxels (DVARS) ^4^, which quantified the mean change in image intensity between the time points. We used the DVARS and six rigid motion parameters for the preprocessed fMRI time-series to regress out the effects of head motion. Subsequently, time series was extracted from the white matter and cerebrospinal fluid, and those time-series were regressed out from preprocessed fMRI data to control for the effect of physiological noise.

# *Regions of interest*

# The VTA, core of the NAc, shell of the NAc, and mPFC masks were created as the region of interests (ROI)s (Fig. 1a). The VTA mask was developed by creating a 3-mm-radius of a sphere centered on the center of the published putative VTA template ([x, y, z] = [−2, −20, −18]) ^5^. Published putative core (pCore) and shell (pShell) templates ^6^ were used to create core and shell masks. To create the right core mask, a 3-mm radius of a sphere was created centered on the middle of the right pCore ([x, y, z] = [12, 12, -12]). Then, the left core mask was developed by creating a 3-mm radius of a sphere centered on the left counterpart of the right pCore ([x, y, z] = [-12, 12, -12]). Right and left core masks were merged to create the core mask. To create the right shell mask, a 3-mm radius of a sphere was created centered on the middle of right pShell ([x, y, z] = [12, 8, -12]). Then, the left shell mask was developed by creating a 3-mm radius of a sphere centered on the left counterpart of the right pShell ([x, y, z] = [-12, 8, -12]). Right and left shell masks were merged to create the shell mask. Based on our previous report ^7^, the PFC region in the medial superior frontal cortex, which showed a difference in resting-state functional connectivity with the VTA across various psychiatric disorders including SCZ, MDD, and bipolar disorder, was adapted for the mPFC mask. A 5-mm radius of a sphere centered on the peak voxel of cluster in the superior frontal lobe (the Brodmann area 8) ([x, y, z] = [6, 34, 50]) was created for the mPFC mask. Subsequently, the averaged first principal component of the time-series from all voxels included in the ROIs was extracted for the DCM analysis. To avoid time-series extraction from the non-brain region, each ROI mask was multiplied with the binarized whole brain mask thresholded at 0.5 of the intensity.

# *Parametric empirical bayes estimation*

# We conducted group-level inference for spDCM using an empirical Bayesian approach with SPM12 ^8, 9^. Parametric empirical Bayes (PEB) consists of Bayesian model reduction, searching over nested models, and comparison of effective connectivity parameters ^10^. Empirical Bayes refers to fitting of hierarchical models. In hierarchical models, constraints on the posterior density over model parameters at any given level are provided by the level above. These constraints are called empirical priors because they are informed by empirical data ^9^. First, a Bayesian model reduction ^11, 12^, which involved Bayesian inversion and comparison of models that were reduced forms of the fully connected model, was applied. Then, connections between the four ROIs were switched on and off in an iterative process to test their effect on free energy, an approximation of the log model evidence ^9^. Parameters that did not contribute to the free energy were switched off by setting their prior mean and variance to zero.

# **Table S1. Clinical assessment per group**

|  | SCZ  (n = 140) | MDD  (n = 127) | ASD  (n = 119) |
| --- | --- | --- | --- |
| PANSS positive | 14.16 ± 5.13  (7.00–29.00)  Missing: 14 |  |  |
| PANSS negative | 16.43 ± 6.32  (7.00–34.00)  Missing: 13 |  |  |
| PANSS general | 30.42 ± 9.17  (16.00–56.00)  Missing: 13 |  |  |
| PANSS total | 58.85 ± 20.81  (0.00–114.00)  Missing: 10 |  |  |
| BDI-II |  | 25.09 ± 10.58  (0.00–47.00)  Missing: 21 |  |
| AQ social skill deficits |  |  | 7.69 ± 2.13  (1.00–10.00)  Missing: 8 |
| AQ attention switching problems |  |  | 7.40 ± 1.84  (0.00–10.00)  Missing: 8 |
| AQ attention to detail |  |  | 5.48 ± 2.18  (1.00–10.00)  Missing: 8 |
| AQ communication difficulties |  |  | 7.42 ± 1.98  (1.00–10.00)  Missing: 8 |
| AQ imagination deficits |  |  | 6.50 ± 1.93  (3.00–10.00)  Missing: 8 |
| AQ total |  |  | 34.47 ± 5.99  (14.00–50.00)  Missing: 8 |

SCZ, schizophrenia; MDD, major depressive disorder; ASD, autism spectrum disorders; PANSS, Positive and Negative Syndrome Scale; BDI-II, Beck Depression Inventory-II; AQ, Autism-Spectrum Quotient Test

# **Table S2. Image acquisition parameters per procedure**

| Site | COI | KTT | KUT | SWA | UTO |
| --- | --- | --- | --- | --- | --- |
| MRI scanner | Siemens verio | Siemens Trio | Siemens TimTrio | Siemens verio | GE MR750w |
| Magnetic field strength | 3.0 T | 3.0 T | 3.0 T | 3.0 T | 3.0 T |
| Number of channels per coil | 12 | 8 | 32 | 12 | 24 |
| Field of view (mm) | 212 × 212 | 256 × 192 | 212 × 212 | 212 × 212 | 212 × 212 |
| Matrix | 64 × 64 | 64 × 48 | 64 × 64 | 64 × 64 | 64 × 64 |
| Phase encoding direction | AP | AP | PA | PA | PA |
| Number of slices | 40 | 30 | 40 | 40 | 40 |
| Slice thickness  (mm) | 3.2 | 4.0 | 3.2 | 3.2 | 3.2 |
| Slice gap (mm) | 0.8 | 0 | 0.8 | 0.8 | 0.8 |
| TR (ms) | 2,500 | 2,000 | 2,500 | 2,500 | 2,500 |
| TE (ms) | 30 | 30 | 30 | 30 | 30 |
| Number of volumes | 240 | 182 | 240 | 240 | 240 |
| Total scan time  (min:s) | 10:00 | 6:00 | 10:00 | 10:00 | 10:00 |
| Slice acquisition order | Ascending | Ascending interleaved | Ascending | Ascending | Ascending |

AP, anterior-posterior; PA, posterior-anterior; TR, repetition time; TE, echo time COI, Siemens Verio scanner at the Center of Innovation in Hiroshima University; KTT, a Siemens Trio scanner at Kyoto University; KUT, a Siemens TimTrio scanner at Kyoto University; SWA, a Siemens Verio scanner at Showa University; UTO, GE MR750W scanner at The University of Tokyo Hospital

# **Table S3. Head motion in each group**

|  | HC  (n = 178) | SCZ  (n = 117) | MDD  (n = 127) | ASD  (n = 119) | p-value^*^ |
| --- | --- | --- | --- | --- | --- |
| x (mm) | 0.47 ± 0.37 | 0.36 ± 0.34 | 0.65 ± 0.51 | 0.64 ± 0.45 | < 0.001 |
| y (mm) | 0.57 ± 0.36 | 0.67 ± 0.48 | 0.70 ± 0.40 | 0.76 ± 0.42 | < 0.001 |
| z (mm) | 0.99 ± 0.64 | 0.76 ± 0.49 | 1.09 ± 0.58. | 1.03 ± 0.58 | < 0.001 |

^*^ Kruskal-Wallis rank sum test

HC, healthy controls; SCZ, schizophrenia; MDD, major depressive disorder; ASD, autism spectrum disorders

# **Table S4. Characteristics of male participants**

|  | HC  (n = 110) | SCZ  (n = 77) | MDD  (n = 70) | ASD  (n = 101) | p-value^*^ |
| --- | --- | --- | --- | --- | --- |
| Scanners | COI = 20  KTT = 21  KUT = 24  SWA = 25  UTO = 20 | COI = 0  KTT = 19  KUT = 18  SWA = 14  UTO = 26 | COI = 24  KTT = 0  KUT = 7  SWA = 0  UTO = 39 | COI = 0  KTT = 0  KUT = 0  SWA = 99  UTO = 2 |  |
| Age  (years) | 34.3 ± 9.4  (19.0 - 58.0) | 36.6 ± 9.9  (17.0 - 55.0) | 39.5 ± 10.2  (18.0 - 59.0) | 31.9 ± 8.2  (20.0 - 54.0) | < 0.001 |
| Handedness  (right / left) | 100 / 10 | 70 / 6^**^ | 65 / 5 | 91 / 10 | 0.92 |

^*^ Differences in handedness are tested using a Chi-squared test and in age using a Kruskal-Wallis rank sum test.

^**^ Data missing for one participant.

HC, healthy controls; SCZ, schizophrenia; MDD, major depressive disorder; ASD, autism spectrum disorders; COI, Siemens Verio scanner at the Center of Innovation in Hiroshima University; KTT, a Siemens Trio scanner at Kyoto University; KUT, a Siemens TimTrio scanner at Kyoto University; SWA, a Siemens Verio scanner at Showa University; UTO, GE MR750W scanner at The University of Tokyo Hospital

# **Table S5. Clinical assessment in male participants per group**

|  |  | SCZ  (n = 77) | MDD  (n = 70) | ASD  (n = 101) |
| --- | --- | --- | --- | --- |
|  |  |  |  |  |
| PANSS positive |  | 14.54 ± 5.03  (7.00–26.00)  Missing: 7 |  |  |
| PANSS negative |  | 17.26 ± 6.14  (7.00–31.00)  Missing: 7 |  |  |
| PANSS general |  | 31.03 ± 9.02  (16.00–50.00)  Missing: 7 |  |  |
| PANSS total |  | 59.43 ± 22.65  (0.00–102.00)  Missing: 3 |  |  |
| BDI-II |  |  | 24.36 ± 8.71  (0.00–46.00)  Missing: 11 |  |
| AQ social skill deficits |  |  |  | 7.60 ± 2.13  (1.00–10.00)  Missing: 5 |
| AQ attention switching problems |  |  |  | 7.31 ± 1.85  (0.00–10.00)  Missing: 5 |
| AQ attention to detail |  |  |  | 5.53 ± 2.14  (1.00–10.00)  Missing: 5 |
| AQ communication difficulties |  |  |  | 7.30 ± 1.98  (1.00–10.00)  Missing: 5 |
| AQ imagination deficits |  |  |  | 6.50 ± 1.99  (3.00–10.00)  Missing: 5 |
| AQ total |  |  |  | 5.99 ± 6.11  (14.00–50.00)  Missing: 5 |

SCZ, schizophrenia; MDD, major depressive disorder; ASD, autism spectrum disorders; PANSS, Positive and Negative Syndrome Scale; BDI-II, Beck Depression Inventory-II; AQ, Autism-Spectrum Quotient Test

# **Table S6. Head motion in male participants**

|  | HC  (n = 91) | SCZ  (n = 77) | MDD  (n = 70) | ASD  (n = 101) | p-value^*^ |
| --- | --- | --- | --- | --- | --- |
| x (mm) | 0.49 ± 0.40 | 0.32 ± 0.22 | 0.56 ± 0.43 | 0.67 ± 0.46 | < 0.001 |
| y (mm) | 0. 61 ± 0.33 | 0.65 ± 0.42 | 0.67 ± 0.35 | 0.80 ± 0.44 | 0.002 |
| z (mm) | 1.00 ± 0.66 | 0.77 ± 0.51 | 1.06 ± 0.57 | 1.07 ± 0.60 | < 0.001 |

^*^ Kruskal-Wallis rank sum test

HC, healthy controls; SCZ, schizophrenia; MDD, major depressive disorder; ASD, autism spectrum disorders


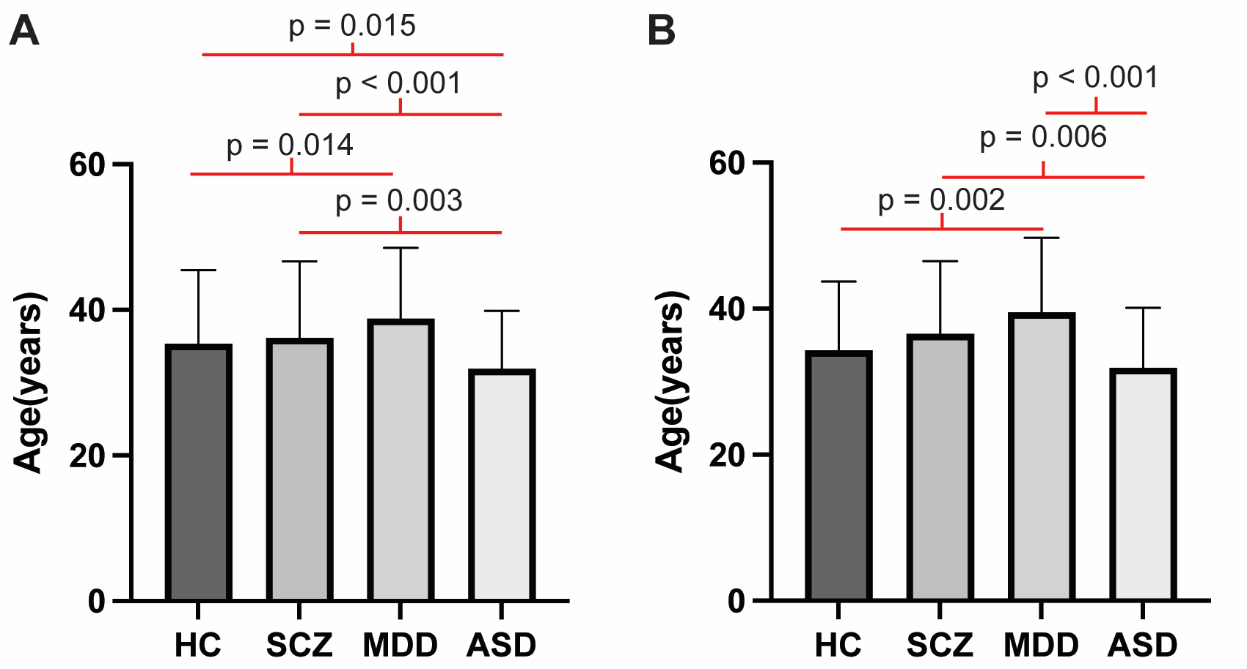


# **Figure S1. Age of all participants (A) and male participants (B)**

Bars depict the mean age in each group, and error bars indicate the standard deviations in each group. P-values are Bonferroni corrected.

Abbreviations: HC, healthy controls; SCZ, schizophrenia; MDD, major depressive disorder; ASD, autism spectrum disorders

# **
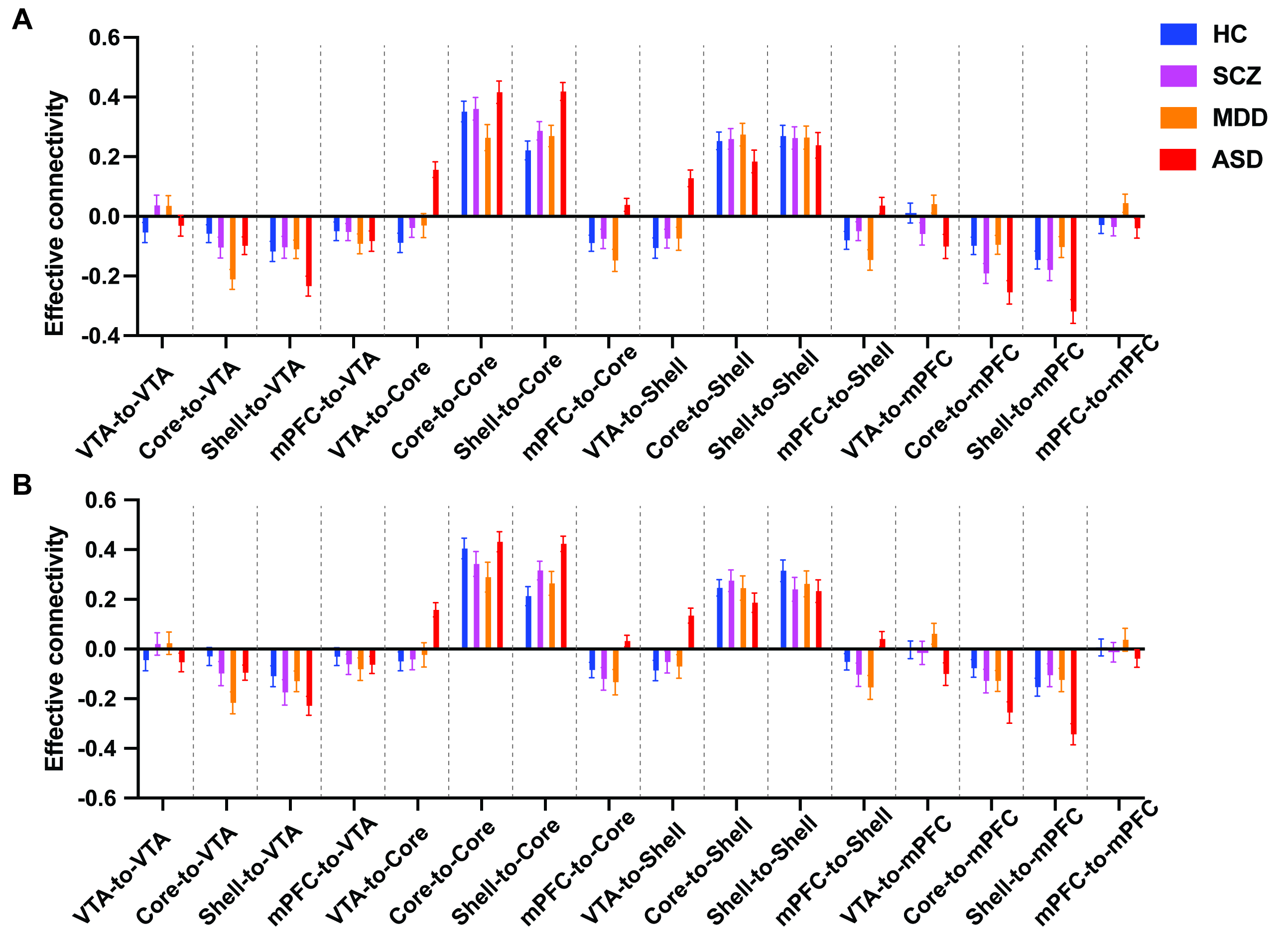
Figure S2. Effective connectivity of all participants (A) and male participants (B)**

Bars depict the mean effective connectivity in each group.

Abbreviations: HC, healthy controls; SCZ, schizophrenia; MDD, major depressive disorder; ASD, autism spectrum disorders; VTA, ventral tegmental area; mPFC, medial prefrontal cortex

**References**

1. Tanaka SC, Yamashita A, Yahata N et al. A multi-site, multi-disorder resting-state magnetic resonance image database. *Scientific Data* 2021; **8**: 227.

2. Yamashita A, Yahata N, Itahashi T et al. Harmonization of resting-state functional MRI data across multiple imaging sites via the separation of site differences into sampling bias and measurement bias. *PLoS Biol* 2019; **17**: e3000042.

3. Andersson JLR, Hutton C, Ashburner J, Turner R, Friston K. Modeling Geometric Deformations in EPI Time Series. *NeuroImage* 2001; **13**: 903-919.

4. Power JD, Mitra A, Laumann TO, Snyder AZ, Schlaggar BL, Petersen SE. Methods to detect, characterize, and remove motion artifact in resting state fMRI. *NeuroImage* 2014; **84**: 320-341.

5. Nakamura Y, Okada N, Kunimatsu A, Kasai K, Koike S. Anatomical Templates of the Midbrain Ventral Tegmental Area and Substantia Nigra for Asian Populations. *Frontiers in Psychiatry* 2018; **9**.

6. Baliki MN, Mansour A, Baria AT et al. Parceling human accumbens into putative core and shell dissociates encoding of values for reward and pain. *J Neurosci* 2013; **33**: 16383-93.

7. Nakamura Y, Okada N, Koshiyama D et al. Differences in Functional Connectivity Networks Related to the Midbrain Dopaminergic System-Related Area in Various Psychiatric Disorders. *Schizophrenia Bulletin* 2020; **46**: 1239-1248.

8. Friston K, Zeidman P, Litvak V. Empirical Bayes for DCM: A Group Inversion Scheme. *Front Syst Neurosci* 2015; **9**: 164.

9. Friston KJ, Litvak V, Oswal A et al. Bayesian model reduction and empirical Bayes for group (DCM) studies. *Neuroimage* 2016; **128**: 413-431.

10. Zeidman P, Jafarian A, Seghier ML et al. A guide to group effective connectivity analysis, part 2: Second level analysis with PEB. *NeuroImage* 2019; **200**: 12-25.

11. Friston K, Penny W. Post hoc Bayesian model selection. *Neuroimage* 2011; **56**: 2089-99.

12. Friston KJ, Li B, Daunizeau J, Stephan KE. Network discovery with DCM. *Neuroimage* 2011; **56**: 1202-21.
